# 67 Double impact: hypophosphatemic rickets and vitamin D intoxication

**DOI:** 10.1093/rheumatology/keac496.063

**Published:** 2022-10-07

**Authors:** N Boutrid, H Rahmoune, H Boutrid, B Bioud, M Amrane

**Affiliations:** 1 LMCVGN Laboratory, Faculty of Medicine, Sétif-1 University, Algeria; 2 Pediatrics, Sétif University Hospital, Algeria; 3 Faculty of Medicine, Algiers-1 University, Algeria; 4 Gynecology-Obstetrics, Bab El-Oued University Hospital, Algiers, Algeria

## Abstract

**Background:**

Vitamin D prevents deficiency rickets, but its prescription must be weighed carefully!

**Objective**

We report a rare case of iatrogenic toxic hypervitaminosis D in a child with vitamin D resistant rickets…!

**Methods:**

We admitted a two-year-old girl, presenting signs of rickets resistant to the usual then therapeutic doses of vitamin D3. Vitamin D2 in oral suspension is then ordered in addition, for a few weeks until the onset of hearing loss motivating her hospitalization in pediatrics.

**Results:**

Biological and radiological explorations revealed renal failure with threatening arterial hypertension, as well as diffuse and severe arteriosclerosis on Doppler signal.

Eviction (stopping vitamin D in all its forms), symptomatic and conservative management by (converting-enzyme inhibitors) and hydration allowed a progressive blood pressure normalisation, arteriosclerosis disappearance (undetected by Doppler signal and by angio-scan) along with a gradual normalising of the kidney function; all this over a period of 2 years

The girl actually had hypophosphatemic rickets, and oral phosphorus (syrup) was also prescribed

**Conclusion:**

Vitamin D is potentially toxic with sometimes severe renal risks (lithiasis, nephrocalcinosis), and hypercalcemia might be severe, especially if renal function is impaired.

Blindly treating any rickets with vitamin D can be adventurous and might expose the child to serious morbidity.

